# Weight Management Advice for Clients with Overweight or Obesity: Allied Health Professional Survey

**DOI:** 10.3390/healthcare4040085

**Published:** 2016-11-14

**Authors:** Suzanne J. Snodgrass, Maya Guest, Ashley K. Kable, Carole James, Samantha E. Ashby, Ronald C. Plotnikoff, Clare E. Collins

**Affiliations:** 1 School of Health Sciences, Faculty of Health and Medicine, University of Newcastle, Callaghan, NSW 2308, Australia; maya.guest@newcastle.edu.au (M.G.); carole.james@newcastle.edu.au (C.J.); samantha.ashby@newcastle.edu.au (S.E.A.); clare.collins@newcastle.edu.au (C.E.C.); 2 Priority Research Centre for Physical Activity and Nutrition, University of Newcastle, Callaghan, NSW 2308, Australia; ron.plotnikoff@newcastle.edu.au; 3 School of Nursing and Midwifery, Faculty of Health and Medicine, University of Newcastle, Callaghan, NSW 2308, Australia; ron.plotnikoff@newcastle.edu.au; 4 School of Education, Faculty of Education and Arts, University of Newcastle, Callaghan, NSW 2308, Australia

**Keywords:** health behaviour, diet, obesity, health promotion, physical activity, allied health, weight management

## Abstract

The prevalence of obesity is increasing. The potential for allied health professionals to intervene through the provision of lifestyle advice is unknown. This study aimed to determine the knowledge, attitudes and practices of health professionals in the provision of dietary and physical activity advice for clients with overweight or obesity. Dietitians, exercise physiologists, nurses, occupational therapists, physiotherapists and psychologists (*n* = 296) working in New South Wales were surveyed using paper-based and online methods. The majority of health professionals (71%) believed that providing weight management advice was within their scope of practice; 81% provided physical activity advice but only 57% provided dietary advice. Other than dietitians, few had received training in client weight management during their professional qualification (14%) or continuing education (16%). Providing dietary advice was associated with: believing it was within their scope of practice (OR 3.9, 95% CI 1.9–7.9, *p* < 0.01), training during their entry-level qualification (OR 7.2, 3.2–16.4, *p* < 0.01) and having departmental guidelines (OR 4.7, 2.1–10.9, *p* < 0.01). Most health professionals are willing to provide lifestyle advice to clients with overweight or obesity but few have received required training. Developing guidelines and training for in client weight management may potentially impact on rising obesity levels.

## 1. Introduction

The prevalence of being overweight or obese has increased substantially over the past decade [1]. In one of the few population-based studies with objective body mass index (BMI) measurement of over 11,000 adults, 67% of men and 52% of women over 24 years of age were overweight (BMI > 25) or obese (BMI > 30) in 1999–2000 [2]. Additionally, adults are progressing from being overweight to being obese or morbidly obese at alarming rates [3,4,5]. Obesity increases the risk of developing chronic diseases including type 2 diabetes, cardiovascular disease and some cancers, with the primary recommendation from the World Cancer Research Fund for adults to be as lean as possible, without becoming underweight [6,7]. A high prevalence of obesity increases the demands on the public health system and on health professionals whose role it is to provide weight loss interventions.

Consequently, there is a clear need for developing strategies to target individuals who are overweight or obese and to prevent further weight gain during a range of patients’ encounters within the health system. Furthermore, although guidelines for providing weight management advice exist, they are not being routinely utilised by health professionals [8]. Within the healthcare setting, weight loss interventions are usually provided by dietitians and general practitioners [9,10,11]. However, other allied health professionals such as physical therapists, occupational therapists, psychologists, nurses, exercise physiologists and occupational health nurses may also be able to provide some evidence-based healthy lifestyle advice to their clients, within their existing role, and potentially before severe co-morbidities develop in clients who may be overweight [12,13,14]. What is not known is whether allied health professionals consider the provision of healthy lifestyle advice as part of their role, whether they have adequate knowledge to provide evidence-based weight management advice, and whether they are already doing this.

Previous studies regarding weight management beliefs and practices of allied health professionals have primarily focused on dietitians [9,15], nurses [16] and physical therapists [17,18]. Surveys of nurses and physical therapists indicate that they believe the provision of weight loss advice is an important component of their client management [17,18,19], and that additional training to improve skills and confidence in providing healthy lifestyle advice would be beneficial. The Counterweight programme [16] in the UK demonstrated that after a six to eight-hour training program, general practice nurses could effectively lead practice-based weight loss interventions that included patient assessment and strategies for healthy eating, physical activity and behaviour change. These patient interventions were provided over six individual or group sessions with continued follow-up and demonstrated that after 12 months, 34% of patients had achieved a weight loss of five percent or more [16]. These results suggest that allied health professionals, in addition to dietitians, may be able to contribute to interventions to effectively assist clients with weight management advice. We have previously reported on educational and personal factors associated with allied health practitioner’s health promotion actions and behaviours in the provision of weight management advice to clients within in the Community Healthy Adult Project (CHAP), a cross-sectional survey across a broad range of allied health professionals (dietitians, physical therapists, exercise physiologists occupational therapists, community nurses, occupational health nurses and psychologists) [20]. To date, however, the views of allied health professionals in regard to provision of weight management interventions have only been reported for individual allied health disciplines without comparisons between disciplines. Further, no study has been identified that includes a range of allied health professionals working in the same setting and region. This enables comparisons of health professional practices and service provision within a single geographic population.

Therefore the aim of the current study was to determine from the CHAP survey in a broad range of Australian allied health professionals their knowledge, attitudes and practices in relation to the provision of healthy lifestyle to clients with overweight or obesity, specifically nutrition and physical activity advice for weight management. In addition, the factors influencing the provision of that advice will be explored.

## 2. Materials and Methods

### 2.1. Study Population

The CHAP study was a cross-sectional survey of allied health professionals working in either privately owned practices or in government-funded hospitals and community health services within the Hunter New England region of New South Wales, Australia [20]. This region encompasses a wide geographical area including metropolitan, regional and rural areas. Recruitment was stratified by seven disciplines: dietitians, physical therapists, exercise physiologists, occupational therapists, community nurses, occupational health nurses and psychologists (Table 1). These disciplines were specifically selected as they were likely to have the capacity to provide brief healthy lifestyle advice, or messages related to diet or physical activity, as part of their routine patient care. Allied health professionals were contacted using either postal mail, email or through their department supervisors.

In order to maximise the response rate, each potential health professional received an announcement (via postal mail or email) to advise them that they may be invited to participate in the study and would soon receive a questionnaire. One week later, allied health professionals were either sent a paper-based survey with a pre-addressed reply paid envelope, or emailed a link to an online survey tool (SurveyMonkey™, SurveyMonkey, Sydney, Australia). All allied health professionals also received an information statement about the study and a letter inviting them to participate. Four weeks after being sent the questionnaire, a thank you/reminder postcard or email was sent to all allied health professionals as a reminder to participate. In total, 81% of correspondence with allied health professionals was via email, and the remainder (19%) was via postal mail. Email was used where allied health professionals had professional email addresses, and postal mail was used to individually contact allied health professionals within larger hospital departments where staff did not have access to individual professional email accounts. As this was an exploratory survey, sample size calculations were not performed a priori. Ethical approval for the study was obtained from by the Human Research Ethics Committees of the Hunter New England Area Health Service and the University of Newcastle, Australia, with participant informed consent implied upon completion of the questionnaire (ethical approved project identification code: H-2010-1010).

### 2.2. Study Questionnaire

The study used a study-specific questionnaire that was developed based on previously published studies of allied health professional’s provision of dietary and healthy lifestyle advice [9,21,22] and on the framework by Godin and colleagues [23], which categorises factors that potentially influence health professional behaviour. From the Godin et al. framework [23], the study questionnaire addressed the following factors: social influences, role and identity, beliefs about capabilities, and behaviours. In addition, general demographic and workplace information was collected to determine characteristics of allied health professionals. Categories used for participants’ characteristics are listed in Table 2. The location category was based on the employer postal code and using the Australian Standard Geographic Classification (ASGC) system and associated Accessibility/Remoteness Index of Australia (ARIA) released by the Australian Bureau of Statistics (ABS) in 2001. The remoteness categories were subsequently collapsed to represent major city, inner regional, outer regional and remote areas [24].

The draft survey questionnaire was tested with an expert panel of academics (*n* = 9) and practicing allied health professionals (*n* = 8) and then modified in response to the feedback given. The questionnaire asked study participants about whether they provided advice on diet or physical activity to clients with overweight or obesity as part of their usual management. They were asked to describe the interventions currently used and factors associated with the provision of advice including previous training, departmental guidelines and health professional beliefs. Free text responses were allowed for describing interventions provided and subsequently categorised into groups of the most common responses.

### 2.3. Statistical Analysis

The two outcomes of interest for this study were whether allied health professionals provided: (1) dietary advice and (2) advice on increasing physical activity. Health professional and workplace characteristics and potential confounders (gender, highest level of qualification, area of practice, location of practice, years in clinical practice, hours worked per week, number of new clients per week, proportion of clients overweight or obese, number of referrals made to other services for weight management, department guidelines for providing weight management advice, having received education in weight management during their entry-level professional qualification, and continuing education in weight management) were compared across types of health professional groups using Pearson’s chi-square test and analysis of variance. Percentages were used to indicate the proportions of health professionals providing dietary and physical activity advice in various formats (i.e., printed or online material, or client discussion). Logistic regression models were used to determine if health professional group, attitudes, and workplace factors were associated with the provision of (1) dietary or (2) physical activity advice. All variables of interest were initially included in the models, and backwards stepwise elimination was used to exclude variables which were not significant at the *p* = 0.1 level based on the Wald Test. Odds ratios with 95% confidence intervals (CI) are reported. The statistical analysis package STATA V11.1 (StatCorp, College Station, TX, USA) was used for analysis [25].

## 3. Results

### 3.1. Participant Characteristics

Although the exact number of health professionals working in the Hunter New England region could not be confirmed, of the estimated 1493 allied health professionals invited to participate, 296 responded (response rate 20%). The composition of the study sample and response rates by health professional group are reported are in Table 1. As there were only seven exercise physiologists and seven occupational health nurse respondents, the exercise physiologists were combined with physical therapists and the occupational health nurses were combined with nurses for the statistical analyses. A total of 37 participants did not complete the questionnaire and were excluded from the analysis; further, some participants from each profession failed to complete a small number of questions, therefore totals in each analysis vary accordingly.

Table 2 reports the distribution of characteristics across the five professional groups. Significant differences existed between the professional groups for the majority of demographic and workplace characteristics. Participants from most health professional groups were predominantly female, with the exception of physical therapists, where 42% were male. Among the surveyed health professionals, approximately half (50%) had postgraduate qualifications with the remaining having undergraduate qualifications. A greater percentage of nurses (79%), and psychologists (81%) had postgraduate qualifications compared to dietitians (42%), physical therapists (35%), and occupational therapists (18%).

### 3.2. Education

As previously reported, 29% of allied health professionals reported receiving education in weight management for clients with overweight or obesity as part of their initial entry-level professional qualification [20], while 12% reported that they were unsure (Table 2). All dietitians had received training in client weight management (100%) during their initial qualification, compared to 26% of physical therapists, 8% of occupational therapists, and 22% of nurses. No psychologists reported receiving weight management training during their entry-level professional qualification. The majority of dietitians (84%) also reported they had attended continuing education or professional development activities related to weight management since completing their entry-level qualifications. In contrast, only 19% of physical therapists and exercise physiologists, 16% of occupational therapists, 20% of nurses, and 20% of psychologists reported such professional development.

### 3.3. Weight Management and Physical Activity Interventions

Table 3 reports results related to the provision of dietary advice summarised into categories from the free text responses. We have previously reported that the most common strategy used by health professionals in providing dietary advice was related to energy balance, portion control, or reducing total energy/caloric intake [20]. The most common formats in which advice was provided were via discussion with clients (93%) and printed information (62%).

Table 4 shows the results of both univariate logistic regression modelling and the parsimonious model on factors associated with allied health professionals providing dietary advice. All surveyed dietitians provided dietary advice and were therefore removed from the model in these analyses. The modelling indicated that believing it was within their scope of practice (OR 3.9, 95% CI 1.9–7.9, *p* < 0.01), having received weight management education within their entry-level qualification (OR 7.2, CI 3.2–16.4, *p* < 0.01), and having departmental or discipline practice guidelines (OR 4.7, CI 2.1–10.9, *p* < 0.01) significantly increased the odds of allied health professionals reporting they provided dietary advice to their clients who were overweight or obese. A high R^2^ (0.25) for the parsimonious model confirms that a high proportion of variability in whether or not a health professional in this study population provided dietary advice is accounted for by these factors.

The majority (81%) of participants reported providing physical activity advice (Table 5), and most provided this advice via discussion with their clients (94%). We have previously reported that the most common advice provided is to recommend a general increase in activity, particularly walking [20], reported by 56% of participants.

These results indicate that believing it was within their scope of practice (OR 2.7, 95% CI 1.1–6.0, *p* = 0.03) and having attended continuing education in weight management (OR 7.4, CI 1.3–40.5, *p* = 0.02) significantly increased the odds of allied health professionals reporting that they provided physical activity advice to their clients who were overweight or obese (Table 6). Health professional discipline was also a significant factor in the model (OR 0.2, CI 0.04–0.8), *p* = 0.02). A high R^2^ (0.31) for the parsimonious model confirms that a high proportion of variability in whether or not health professionals in this study population provided physical activity advice is accounted for by these factors.

## 4. Discussion

The current study demonstrates that the majority of health professionals believe it is within their scope of practice to provide dietary and physical activity advice for weight management. Within each discipline surveyed, ≥60% believed it was within their scope of practice, with the exception of occupational therapists, where this was 47%. However, in this survey few health professionals other than dietitians reported having received any education or training in provision of weight management advice. Health professionals in the current survey were more likely to provide dietary and physical activity advice if they believed it was within their scope of practice and if they had received education in client weight management either during their entry-level professional qualification or by attending continuing education. This indicates that allied health professionals, in addition to dietitians who commonly spend large proportion of their time in provision of weight management services [9,10,11], may be responsive to receiving further training and continuing education in the area of weight management as a strategy to assist their clients who are overweight or obese. Further, the inclusion of weight management education within entry-level curricula may encourage more health professionals to provide advice for weight management, having an impact on rising levels of obesity.

The results of this survey suggest that health professionals are potentially willing and able to provide an initial basic intervention to clients earlier in their weight gain trajectory through provision of evidence-based information on making lifestyle changes. Indeed, one pilot study has shown that dental hygienists can successfully provide a healthy weight intervention to children as part of their routine care [26]. Provision of consistent weight management messages by a wide range of allied health professionals could potentially ease the burden on the dietetics profession and reserve their expertise for those clients requiring specialised dietetics interventions to address co-morbidities associated with existing chronic conditions. Further research is now needed to examine whether a range of allied health professionals, who are willing to contribute to the team providing weight management advice, can impact on the weight gain trajectories of their clients and potentially impact on obesity prevalence.

Few of the allied health professionals in the current survey had received any education or training related to weight management, despite the high numbers believing it was within their scope of practice to provide this advice and the high numbers already providing both dietary and physical activity advice for weight management. This is in agreement with a previous survey reporting that, although health professionals usually promote the concepts of lifestyle modification, few had adequate knowledge for providing practical advice on diet or physical activity [27]. For those who did report receiving education in weight management in the current study, it is unclear how much of this included dietary advice versus physical activity advice, or the source and quality of this instruction. For those who were providing advice without having received weight management education, the quality of the advice given is unclear. With the high percentage of allied health professionals willing and able to provide weight management advice, it is essential that adequate education programs and evidence-based resources be developed [27].

In the current survey, having had previous education in client weight management was associated with health professionals providing both dietary and physical activity advice to clients (Table 4 and Table 6). Having previous education may increase a health professional’s confidence in providing weight management advice, leading to greater participation of health professionals in the management of obesity. Indeed, one previous survey of health professionals found that self-efficacy was the best predictor for providing healthy lifestyle advice to clients [17]. As self-efficacy can be a mediator of the provision of advice to clients, future research should consider measuring it. If self-efficacy is lacking in health professionals, future research could investigate methods to improve self-efficacy within training programs for the provision of lifestyle advice.

Interestingly, a health professional’s area of practice (e.g., community care, public hospital) and their location of practice (e.g., major city, regional) were not associated with whether or not they provided advice. These factors differed between health disciplines (Table 2) suggesting that variability in these factors may have possibly contributed to their non-significant result in the regression modelling. This was despite a significant association between health professional discipline and the provision of advice. A health professional’s gender was not associated with the provision of dietary or physical activity advice. However, the discipline with the highest percentage of male respondents was physical therapists. Therefore it is possible that gender may have had some influence on the responses of this group or the findings specific to physical therapists, as compared to the other health professional disciplines. The impact of gender on the provision of advice is an area for future study, and would require a greater proportion of male respondents than observed in the current study. Nonetheless, the representation of gender within the disciplines in the current study reflects the gender balance within these disciplines in the clinical setting.

An important factor associated with health professionals providing both dietary and physical activity advice for weight management was their belief that it was within their scope of practice. This suggests that continuing education in weight management and the provision of evidence-based advice will be required, if health professionals’ beliefs about their role in the provision of care are to be influenced. Having departmental guidelines was associated with the provision of dietary, and to a lesser extent physical activity advice. However, few health professional disciplines in the current study, other than dietitians, reported having departmental guidelines for provision of dietary or physical activity advice. The development and implementation of guidelines that would be applicable and acceptable to a wide range of allied health professionals may facilitate provision of dietary and physical activity advice by these professionals, and this is an area for future research.

Despite the fact that the minority of allied health professionals, with the exception of dietitians, had received any education or training in the provision of healthy lifestyle or weight management advice for clients with overweight or obesity, the majority believed it was within their scope of practice and many reported already providing this type of advice to their clients. This indicates that education programs in weight management are urgently needed so that allied health professionals can provide consistent and evidence-based weight loss advice, even if brief. Future research is needed to determine the types of education formats that would be acceptable and engaging for health professionals, and whether this differs for different health professional disciplines, age groups or genders.

### Limitations

This study is limited to a single geographical region in Australia, and to the health professional groups surveyed. Though the sample was considered representative of allied health professionals in these groups within Australia, including additional health professional groups or those from other countries or regions may produce different results. Furthermore, it was not possible to evaluate the quality of weight management advice provided by health professionals, as the survey relied on the health professionals’ self-report.

## 5. Conclusions

In conclusion, a wide range of allied health professionals are willing to provide weight management advice to clients who are overweight or obese and many are already providing dietary and physical activity advice for weight management, yet few have received any evidence-based training in the provision of such advice. These results provide a background for the development of guidelines for allied health professionals for providing weight management and healthy lifestyle advice, which may improve their management of clients who are overweight or obese. The future implementation of these guidelines may impact the obesity crisis and its burden on the health system.

## Figures and Tables

**Table 1:** Composition of the study sample, by allied health profession.

Type of Health Professional	Invited	Participated (*n*) *	Response Rate (%)
Dietitians	129	42	32.6
Physical therapists	272	65	23.9
Occupational therapists	209	57	27.3
Psychologist	200	26	13.0
Exercise physiologists	30	7	23.3
Occupational health nurses	23	7	30.4
Nurses	630	92	14.6
TOTAL	1493	296	19.8

* *n* represents the total number of respondents for each health professional group.

**Table 2:** Demographics of allied health professionals (*n* = 296) participating in survey.

Variable	Dietitian *n* (%), *n* = 36	Physical Therapist *n* (%), *n* = 72	Occupational Therapist *n* (%), *n* = 51	Nurse *n* (%), *n* = 79	Psychologist *n* (%), *n* = 21	Total *n* (%) *, *n* = 259	Pearson Chi^2^		
							Chi^2^	*df*	*p*
Gender									
Male	2 (5.6)	30 (42)	5 (9.8)	7 (8.9)	4 (15)	48 (19)			
Female	34 (94)	42 (58)	46 (90)	72 (91)	17 (81)	211(81)	37.0	4	<0.01
Highest level of qualification									
Undergraduate	21 (58)	47 (65)	42 (82)	16 (21)	4 (19)	130 (50)			
Postgraduate	15 (42)	25 (35)	9 (18)	62 (79)	17 (81)	128 (50)	64.2	4	<0.01
Area of practice									
Aged, community, home care	15 (42)	2 (2.8)	16 (31)	35 (44)	13 (62)	81 (31)			
Rehabilitation centre	0 (0.0)	4 (5.6)	7 (14)	1 (1.3)	1 (4.8)	13 (5.0)			
Public hospital	10 (28)	27 (38)	18 (35)	32 (41)	3 (14)	90 (35)			
Private practice	11 (31)	37 (51)	5 (9.8)	3 (3.0)	2 (7.7)	58 (20)			
Other including mental health	0 (0)	2 (2.8)	5 (9.8)	8 (10)	2 (9.5)	17 (6.6)	98.3	16	<0.01
Location of practice									
Major City	10 (28)	37 (51)	22 (43)	16 (20)	13 (62)	98 (38)			
Inner Regional	7 (19)	6 (8.3)	11 (22)	15 (19)	3 (14)	42 (16)			
Outer Regional	16 (44)	23 (32)	15 (29)	38 (48)	5 (24)	97 (37)			
Remote	3 (8.3)	3 (4.2)	2 (3.9)	10 (11)	0 (0.0)	17 (6.6)			
Unreported	0 (0)	3 (4.2)	1 (2.0)	1 (1.3)	0 (0)	5 (1.9)	32.9	16	<0.01
Within scope of practice to provide advice									
Yes	34 (94)	60 (83)	23 (47)	53 (67)	12 (60)	182 (71)			
No	2 (5.6)	12 (17)	26 (53)	26 (33)	8 (40)	74 (29)	30.5	4	<0.01
Training in weight management during entry-level professional qualification									
Yes	38 (100) *	19 (26)	4 (8)	17 (22)	0 (0.0)	76 (29)			
No	0 (0)	43 (60)	39 (76)	53 (67)	18 (86)	153 (59)			
Unsure	0 (0)	10 (14)	8 (16)	9 (11)	3 (14)	30 (12)	110	8	<0.01
Attended continuing education in weight management	32 (84)	14 (19)	9 (16)	18 (20)	5 (20)	78 (28)	71.5	4	<0.01
Department has guidelines for providing weight management advice	20 (57)	10 (14)	5 (10)	27 (35)	4 (20)	66 (26)	32.2	4	<0.01
Years in clinical practice	11 (9)	14 (10)	10 (7)	22 (9)	11 (6)	15 (10)	19.7	4	<0.01
Hours worked per week	31 (10)	35 (11)	37 (9)	36 (9)	31 (7)	35 (10)	3.2	4	0.02
Number of new clients/week	7 (4)	14 (18)	5 (6)	13 (22)	3.0 (3)	10 (16)	4.7	4	<0.01
% clients overweight or obese	58 (30)	47 (25)	38 (23)	42 (26)	27 (21)	44 (26)	6.1	4	<0.01
Number referrals/week made to other services for weight management	8 (11)	11 (24)	7 (20)	10 (27)	2 (7)	9 (22)	0.9	4	0.5 ^†^

* *n* represents the total number of respondents for each health professional group. Percentages are calculated for individual items, as the number of responses per item differed (missing responses were not included in the percentage calculation); ^†^ Non-significant values truncated at one decimal place.

**Table 3:** Allied health professionals reported practices related to provision of dietary advice for weight management.

Variable	Dietitian *n* (%)	Physical Therapist *n* (%)	Occupational Therapist *n* (%)	Nurse *n* (%)	Psychologist *n* (%)	Total *n* (%)	Pearson Chi^2^		
							Chi^2^	df	*p*
	*n* = 36	*n* = 70	*n* = 48	*n* = 75	*n* = 19	*n* = 248			
Yes, dietary advice is provided	36 (100)	32 (46)	10 (21)	55 (73)	8 (42)	141 (57)			
No, dietary advice is not provided	0 (0)	38 (54)	38 (79)	20 (27)	11 (58)	107 (43)	66.2	4	<0.01
Format in which dietary advice is provided if dietary advice provided									
Dietary info provided via printed info	36 (100)	9 (28)	5 (50)	34 (62)	4 (50)	88 (62)	38.9	4	<0.01
Dietary info provided via discussion with client	36 (100)	28 (88)	8 (80)	53 (96)	6 (75)	131 (93)	11.6	4	0.02
Dietary info provided via internet/electronic info	18 (50)	2 (6.3)	1 (10)	9 (16)	1 (13)	31 (22)	23.4	4	<0.01

**Table 4:** Logistic regression modelling of factors associated with provision of dietary advice.

		Univariate Analysis			Parsimonious Model		
Variable	Response	Odds Ratio	(95% CI)	*p*-Value	Odds Ratio	(95% CI)	*p*-Value
Providing advice within scope of practice	Yes	5.3	(2.9, 9.7)	<0.01	3.9	(1.9, 7.9)	<0.01
	No	*					
Gender	Female	1.3	(0.7, 2.5)	0.37 ^†^			
	Male	*					
Profession	Occupational therapist	0.3	(0.1, 0.7)	<0.01			
	Nurse	3.3	(1.6, 6.5)	<0.01			
	Psychologist	0.9	(0.3, 2.4)	0.78			
	Physical therapist	*					
Years in practice	<5	*					
	5–9.9	1.0	(0.4, 2.4)	0.99			
	10–19.9	1.2	(0.6, 2.6)	0.63			
	>20	1.5	(0.7, 3.2)	0.28			
Location of practice	Major City	*					
	Inner Regional	0.8	(0.4, 1.7)	0.55			
	Outer Regional	1.0	(0.6, 1.9)	0.90			
	Remote	0.9	(0.2, 2.4)	0.89			
	Unreported	0.2	(0.9, 2.1)	0.23			
Highest level of qualification	Undergraduate	*					
	Postgraduate	1.7	(1.0, 2.8)	0.05			
Hours worked per week	<30	*					
	30–38	1.1	(0.6, 2.2)	0.75			
	>38	0.7	(0.4, 1.4)	0.35			
Weight management education in entry-level professional qualification	No	*					
	Yes	9.8	(4.5, 21.2)	<0.01	7.2	(3.2, 16.4)	<0.01
	Unsure	1.8	(0.7, 4.1)	0.20	2.0	(0.8, 5.2)	0.17
Attended continuing education in weight management	No	*					
	Yes	5.8	(2.9, 11.9)	<0.01			
Departmental guidelines	No	*					
	Yes	6.8	(3.2, 14.6)	<0.01	4.7	(2.1, 10.9)	<0.01

* Indicates reference category for categorical variables; ^†^ Non-significant values truncated at one decimal.

**Table 5:** Allied health professionals reported practices related to provision of physical activity advice for weight management.

Response	Dietitian *n* (%)	Physical Therapist *n* (%)	Occupational Therapist *n* (%)	Nurse *n* (%)	Psychologist *n* (%)	Total *n* (%)
	*n* = 36	*n* = 68	*n* = 46	*n* = 74	*n* = 18	*n* = 242
Yes, physical activity advice provided	35 (97)	62 (91)	30 (65)	60 (81)	10 (56)	197 (81)
No, physical activity advice not provided	1 (2.8)	6 (8.8)	16 (35)	14 (19)	8 (44)	45 (19)
Format in which physical activity advice is provided						
Physical activity information provided—printed information	19 (54)	34 (55)	10 (33)	30 (50)	4 (40)	97 (50)
Physical activity information provided—discussion with client	33 (94)	61 (98)	29 (97)	53 (88)	9 (90)	185 (94)
Physical activity information provided—internet/electronic info	4 (11)	8 (13)	1 (3.3)	5 (8.3)	0 (0)	18 (9.1)

**Table 6:** Logistic regression modelling of factors associated with providing physical activity advice.

		Univariate Analysis			Parsimonious Model		
Variable	Response	Odds Ratio	(95% CI)	*p*-Value	Odds Ratio	(95% CI)	*p*-Value
Providing advice within scope of practice	Yes	8.2	(4.0, 16.7)	<0.01	2.7	(1.1, 6.0)	0.03
	No	*					
Gender	Female	0.5	(0.2, 1.43)	0.21 ^†^			
	Male	*					
Profession	Physical therapist	*					
	Occupational therapist	0.2	(0.1, 0.5)	<0.01	0.3	(0.1, 1.1)	0.08
	Nurse	0.4	(0.2, 1.2)	0.10	0.8	(0.2, 2.7)	0.72
	Psychologist	0.1	(0.0, 0.4)	<0.01	0.2	(0.04, 0.8)	0.02
	Dietitian	3.6	(0.4, 30.8)	0.25	1.0	(0.1, 11.6)	0.98
Years in practice		1.0	(1.0, 1.0)	0.60			
Location of practice	Major City	*					
	Inner Regional	0.7	(0.3, 1.7)	0.37			
	Outer Regional	0.7	(0.3, 1.6)	0.46			
	Remote	0.6	(0.2, 2.0)	0.36			
Area of practice	Aged, community, home care	*					
	Rehabilitation centre	3.2	(0.4, 26.3)	0.29			
	Public hospital	1.1	(0.5, 2.9)	0.87			
	Private practice	1.9	(0.7, 5.3)	0.21			
	Other including mental health	1.4	(0.4, 4.8)	0.55			
Highest level of qualification	Undergraduate	*					
	Postgraduate	1.1	(0.6, 2.2)	0.71			
Hours worked per week		1.0	(1, 1)	0.50			
Hours worked per week in direct patient care		1.0	(1, 1.1)	0.02			
Weight management education in entry-level professional qualification	No	*					
	Yes	5.7	(2.0, 17.0)	<0.01			
	Unsure	1.4	(0.5, 4.1)	0.49			
Attended continuing education in weight management	No	*					
	Yes	11.3	(2.6, 48.0)	<0.01	7.4	(1.3, 40.5)	0.02
Proportion of clients overweight or obese		1.0	(1.0, 1.1)	<0.01	1.0	(1.0, 1.1)	<0.01
Number of clients referred to other services for weight management		1.0	(0.99, 1.0)	0.5	0.99	(0.96, 1.0)	0.02
Departmental guidelines	No	*					
	Yes	3.3	(1.2, 8.8)	0.01			

* Indicates reference category for categorical variables; ^†^ Non-significant values truncated at one decimal.
